# The Future of Cryopreservation in Assisted Reproductive Technologies

**DOI:** 10.3389/fendo.2020.00067

**Published:** 2020-02-20

**Authors:** Ernesto Bosch, Michel De Vos, Peter Humaidan

**Affiliations:** ^1^Instituto Valenciano de Infertilidad, Valencia, Spain; ^2^Centre for Reproductive Medicine, Universitair Ziekenhuis Brussel, Brussels, Belgium; ^3^The Fertility Clinic, Skive Regional Hospital, Skive, Denmark; ^4^Faculty of Health, Aarhus University, Aarhus, Denmark

**Keywords:** oocyte cryopreservation, embryo cryopreservation, freeze-all, elective frozen embryo transfer (eFET), high responders, ovarian hyperstimulation syndrome (OHSS), polycystic ovary syndrome (PCOS), preimplantation genetic testing (PGT)

## Abstract

Societal changes and the increasing desire and opportunity to preserve fertility have increased the demand for effective assisted reproductive technologies (ART) and have increased the range of scenarios in which ART is now used. In recent years, the “freeze-all” strategy of cryopreserving all oocytes or good quality embryos produced in an IVF cycle to transfer later—at a time that is more appropriate for reasons of medical need, efficacy, or desirability—has emerged as an accepted and valuable alternative to fresh embryo transfer. Indeed, improvements in cryopreservation techniques (vitrification) and the development of more efficient ovarian stimulation protocols have facilitated a dramatic increase in the practice of elective frozen embryo transfer (eFET). Alongside these advances, debate continues about whether eFET should be a standard treatment option available to the whole IVF population or if it is important to identify patient subgroups who are most likely to benefit from such an approach. Achieving successful outcomes in ART, whether by fresh or frozen embryo transfer, is influenced by a wide range of factors. As well as the efficiency of IVF and embryo transfer protocols and techniques, factors affecting implantation include maternal aging, sperm quality, the vaginal and endometrial microbiome, and peri-implantation levels of serum progesterone. The safety of eFET, both during ART cycles and on longer-term obstetric and neonatal outcomes, is also an important consideration. In this review, we explore the benefits and risks of freeze-all strategies in different scenarios. We review available evidence on the outcomes achieved with elective cryopreservation strategies and practices and how these compare with more traditional IVF cycles with fresh embryo transfers, both in the general IVF population and in subgroups of special interest. In addition, we consider how to optimize and individualize “freeze-all” procedures to achieve successful reproductive outcomes.

## Introduction

Increasing demand for assisted reproductive technologies (ART) and improvements in cryopreservation techniques are re-shaping the therapeutic landscape in fertility treatment. Indications for ART are expanding as a result of societal changes and increasing desire and opportunity to preserve fertility, for example for “social” reasons in women wishing to improve their chances of conception at an older age, or for medical reasons such as preservation of oocytes prior to cytotoxic anticancer therapy.

Changes in and increased use of ART protocols and procedures have been fueled recently by the development of more efficient ovarian stimulation protocols—for example, modified luteal phase support after gonadotrophin-releasing hormone (GnRH) agonist triggers in GnRH antagonist protocols (1)—and new methods of cryopreservation (vitrification) as an alternative to the more traditional method of slow freezing (2, 3).

These advances, alongside growing understanding of the factors that can affect the outcomes of ART, are leading to continuing improvement in reproductive outcomes. Important factors that influence outcomes of ART include maternal aging (4) and luteal phase progesterone levels (5), as well as newly recognized confounders such as sperm DNA fragmentation (6), and the vaginal and endometrial microbiome (7).

The first human pregnancy from a frozen-thawed embryo was reported in 1983 and the first live birth in 1984 (8, 9). The strategy to cryopreserve all good quality embryos produced in a fresh cycle and to transfer these embryos in subsequent natural or artificially prepared cycles, has been coined a “freeze-all” strategy (10). This strategy, alternatively named “freeze-only,” first appeared in the literature over 20 years ago. The earliest publications described its application in protocols in which implantation is deferred in order to avoid ovarian hyperstimulation syndrome (OHSS) (11, 12), and is increasingly being used in preimplantation genetic testing (PGT) (13). Pioneers of elective cryopreservation and postponed embryo transfer applied the term “segmentation” of *in vitro* fertilization (IVF) treatments, where ovarian stimulation and oocyte/embryo retrieval is disconnected from the subsequent process of embryo transfer (14); a newer term that perhaps better represents the entire process is elective frozen embryo transfer (eFET) (15).

In this article, we will review perspectives on some of the latest advances, strategies, and practices in ART cycles using elective cryopreservation of embryos, and by extension, cryopreservation of oocytes, and whether these developments are likely to bring improvement to IVF outcomes. We will consider how to optimize reproductive outcomes from fresh and frozen embryo transfers, and how to optimize and individualize the “freeze-all” procedure, presenting clinical data demonstrating which patient populations or situations may benefit from this strategy.

## Cryopreservation Today

As a result of improvements in ART, and particularly in the performance of cryopreservation programs, the practice of freezing oocytes or freezing good quality embryos for subsequent FET at a time that is more appropriate for reasons of OHSS prevention, medical issues, efficacy, or desirability has increased dramatically in recent years.

A survey conducted by the European IVF Monitoring (EIM) consortium of the European Society of Human Reproduction and Embryology (ESHRE) between 2010 and 2014 reported a total of 34,705 oocyte cryopreservation cycles across 17 responding countries with available data (out of 34 countries included) (16). Overall, the number of oocyte cryopreservation cycles reported increased continuously during the 5-year period. However, the quality of data is variable and imprecise, highlighting a need for more rigorous national registries designed to collect detailed information on indications, usage, hormonal priming protocols, safety, and efficiency of reproductive cryopreservation of both oocytes and embryos (e.g., yields per cycle and per indication). More recently, in a separate annual survey of European ART data, the EIM group has added a question concerning the use of cryopreserved oocytes in ART cycles (17).

**Figure 1:**
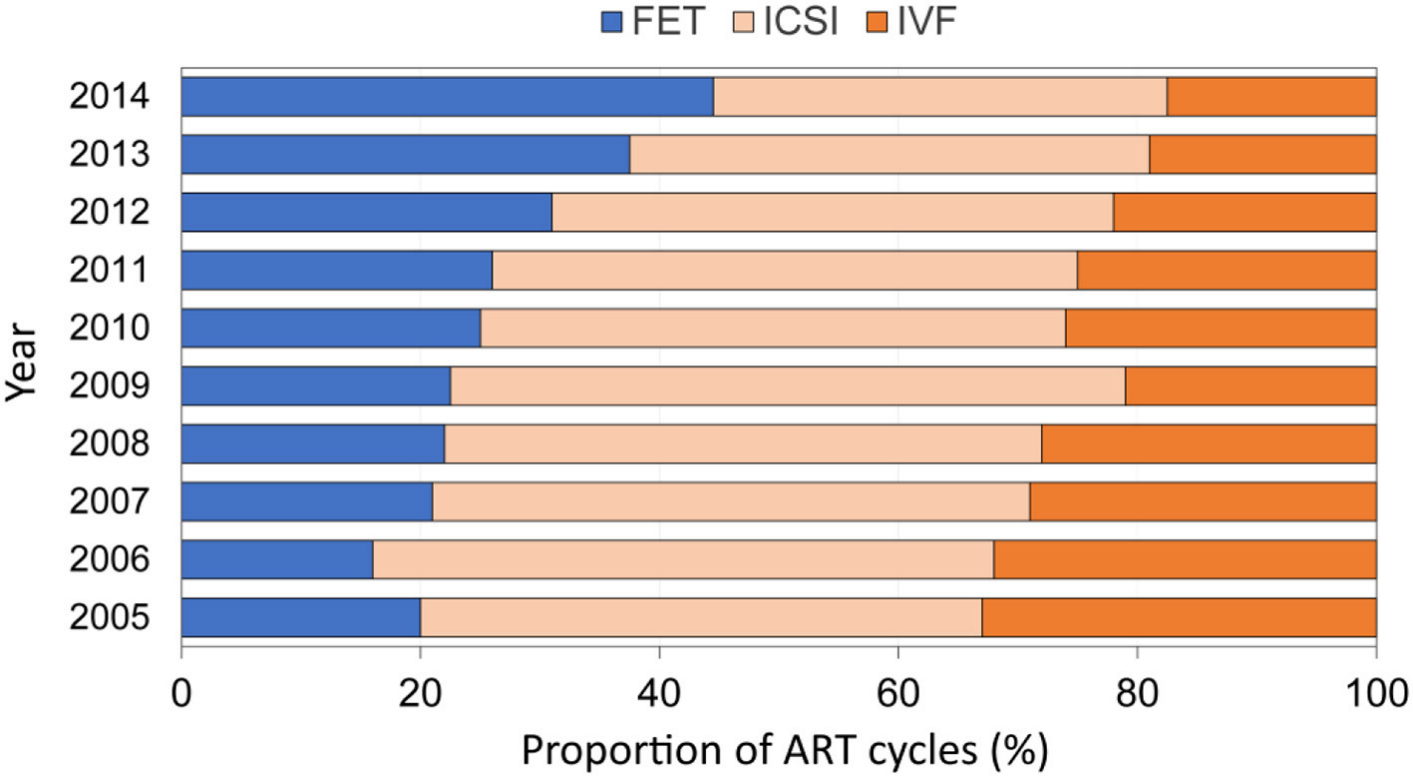
Proportion of FET cycles among all ART cycles reported by year in the United States (2005–2014; US Centers for Disease Control and Prevention). Adapted from Groenewoud et al. (18). FET, frozen embryo transfer; ICSI, intracytoplasmic sperm injection; IVF, *in vitro* fertilization.

European data on embryo cryopreservation cycles are sparse, but data from the US Centers for Disease Control and Prevention show that the proportion of embryo transfers derived from freeze-all cycles is increasing—from around 20% in 2005 to almost 50% in 2014—while the proportion of fresh embryo transfer procedures following IVF and intracytoplasmic sperm injection (ICSI) decreased correspondingly (Figure 1) (18). Editorials published in 2013 proposed that eFET would become accepted as the gold standard practice in IVF (19, 20), although the evidence to support this claim at that time was based mostly on pooled observational data and only limited data from randomized trials. More studies have been conducted recently, and to date, 11 randomized controlled trials comparing eFET and fresh ET have been published (21–31).

### Indications for Cryopreservation in ART Practice

Indications for cryopreservation in ART can be distinguished between elective (patient's choice) and non-elective (medical reasons) (Table 1). Although cryopreservation of embryos or oocytes was originally reserved for women with medical indications and no other fertility options, its use has expanded to include scenarios for elective cryopreservation, most commonly **oocyte donation** (32), and **social oocyte freezing** (33, 34). Patients may also choose to undertake **clinical oocyte freezing**, where a larger batch of oocytes is collected in consecutive ovarian stimulation cycles to increase the opportunities for and likelihood of future IVF success, particularly if there has been recurrent implantation failure (35, 36). Fertility preservation in **transgenders** is another area attracting increasing interest (37, 38). Finally, an option for males who wish to postpone parenthood is an elective sperm cryopreservation at a young age (<40 years) to avoid the age-dependent DNA damage seen in males above the age of 40, which significantly increases the risk of passing age-dependent monogenic and multifactorial diseases on to the offspring (39).

**Table 1:** Indications for cryopreservation in ART practice.

	**Elective**	**Non-elective**
Oocytes	**Oocyte donation, oocyte banking** Avoids the need to match donor's and recipient's cycles, and addresses demand for donor oocytes, thereby alleviating waiting lists (32)	**Medical oocyte freezing** In women about to undergo gonadotoxic treatment for cancer or other conditions, or with a medical pathology that impairs fertility, such as severe endometriosis (41) or genetic conditions including Turner's syndrome (42)
	**Social freezing** Allows women wishing to defer childbearing to preserve their fertility in anticipation of age-related fertility decline (33, 34) Oocyte cryopreservation can provide a feasible alternative where embryo cryopreservation is not an option because of religious, moral or ethical objections, or restrictive legislation (40)	**Incidental oocyte freezing** Emergency freezing in IVF when sperm is not available on the day of oocyte retrieval (43) Storage of “spare” oocytes during IVF (44)
	**Clinical oocyte freezing** Accumulation of oocytes to increase likelihood of future success in cases of poor responders or recurrent implantation failure (35, 36), or to increase their availability for PGT (16)	
	**Transgenders** In the case of female to male change, provides the opportunity to preserve oocytes for future fertilization by a partner or sperm donor (37, 38)	
Embryos	**Preimplantation genetic testing (PGT)** PGT is facilitated by the opportunity to use the freeze-all strategy for storing embryos for transfer in subsequent cycles after testing (27) **Patient's or physician's preference** The ability to store surplus embryos can reduce the number of embryos transferred during a fresh cycle and thus minimize the risk of multiple pregnancy, reduce the need for repeated stimulation cycles, and increase cumulative pregnancy rates (44)	**Elevated progesterone** Elevated progesterone in the late follicular phase has a negative impact on pregnancy rate, although the reasons for this are not entirely clear (45–47) **Avoidance of OHSS** Embryos may be cryopreserved rather than proceeding with a fresh embryo transfer to allow ovarian recovery and thus prevent OHSS when excess follicle development has occurred following ovarian stimulation in the IVF cycle (25, 48)

The use of oocyte cryopreservation tends to avoid the moral objections or legal restrictions that can be associated with embryo cryopreservation and storage, as well as the disputes that can arise if a couple later separates (16), and provides a feasible option for women who do not wish to cryopreserve embryos (40, 44). Nevertheless, embryo cryopreservation is an established technique and there is some evidence from large observational studies that implantation and pregnancy rates are higher from frozen–thawed embryos than when embryos derived from frozen oocytes are used (40, 49). **PGT-A** is the main medical reason for elective embryo cryopreservation (27) and allows time for a suitable euploid embryo to be identified for transfer. Other situations presenting a medical reason for cryopreservation to allow embryo implantation in a later cycle are **elevated progesterone in the late follicular phase** (suggested to have a negative impact on pregnancy rates) (45–47) and **prevention of OHSS**, a potentially life-threatening complication of ovarian stimulation in IVF cycles (25, 48).

There may be various reasons other than medical to prefer eFET as a first-choice strategy in the clinic. Although available published evidence (reviewed later) shows that eFET has better results than fresh embryo transfer only in women with polycystic ovary syndrome (PCOS), high responders, and in the setting of PGT-A (15), FET performs no worse than fresh embryo transfer in normal responders; therefore, there is no need to fear the practice of cryopreservation.

The following sections explore considerations for the use of freeze-all strategies in different scenarios.

## Freeze-All in Art: What Do We Need to Know?

An interesting clinical perspective on the strengths, weaknesses, opportunities, and threats associated with cryopreservation was published recently (50). The growing acceptance that cryopreservation is becoming a substantial part of ART practice raises a number of questions. Is it safe? Are there any long-term health consequences? Is it efficient? Do we need to re-design ovarian stimulation protocols to enhance efficiency compared with fresh embryo transfers? These and other topics are addressed below.

### How Many Oocytes Do We Need?

There is no substitute for having a sufficient number of oocytes. Two large retrospective studies have demonstrated an association between oocyte number and cumulative live birth rate (CLBR) in ART. In fresh embryo transfers, the live birth rate plateaued at a level of 15 oocytes (51), whereas data from vitrification programs showed that CLBR steadily increased with the number of oocytes and no plateau was observed (52). Thus, the higher numbers of oocytes retrieved in high responders will result in higher CLBR. Whether the follicular output rate of a normal responder can be boosted in order to convert a normal responder into a high responder, and whether this will improve CLBR, are more controversial.

The Goldman model for social freezing calculates the probability of live birth for social freezers depending on the age at which they had their oocytes cryopreserved and the number of cryopreserved oocytes, and clearly demonstrates that a higher number of oocytes is very important for efficiency (53). The largest published cohort of freeze-all outcomes is in Chinese women, mostly young patients with tubal pathology, who also had increasing CLBR with higher numbers of oocytes; with ≥16 oocytes, a CLBR of more than 50% was achieved in all age groups after one freeze-all cycle (54).

### How Do We Optimize Mature Oocyte Yield and Oocyte Quality?

The optimum ovarian stimulation protocol for reproductive cryopreservation needs to be safe, convenient, and to achieve maximum ovarian response. This is particularly important for women who only have one chance to cryopreserve their oocytes, for example prior to cancer chemotherapy. Experience from the ART clinic suggests that the GnRH antagonist protocol followed by a GnRH agonist trigger is the shortest, safest, and most convenient stimulation protocol to obtain good quality oocytes and can give a maximum ovarian response in these patients (55).

#### At What Follicle Size Should We Trigger Ovulation to Achieve the Highest Proportion of Good Quality Mature Oocytes?

Data from a non-interventional, retrospective analysis of 165 Vietnamese women show that follicles of between 12 and 19 mm in diameter on the day of GnRH agonist trigger are most likely to yield mature oocytes on the day of oocyte retrieval and to lead to top-quality embryos (56). Balancing optimum follicle size and day of trigger is a critical decision, as a delay in triggering can lead to serum progesterone elevation (56), which is known to reduce implantation rates following fresh embryo transfer (47, 57).

#### What Is the Impact of Elevated Progesterone in the Late Follicular Phase on Oocyte Quality?

Two studies have demonstrated that there is no negative impact from elevated serum progesterone in the late follicular phase on oocyte quality or ongoing pregnancy rates in recipients of donated oocytes (58, 59). However, in a retrospective study of 3,400 cycles, the proportion of transferrable embryos among total embryos decreased as progesterone levels increased, and elevated late follicular-phase serum progesterone had a negative impact on CLBR after fresh ET and FET in IVF patients (60). According to a recent systematic review, elevated follicular fluid levels of progesterone may not be deleterious for oocyte quality (61).

#### Random-Start Controlled Ovarian Stimulation (RSCOS)

In the setting of cryopreservation, where there is no need for a fresh transfer in the same cycle, the cycle phase is not critical. The concept of **“ovarian follicular waves”** (62)—the existence of multiple follicular waves within one menstrual cycle—is more relevant for cryopreservation protocols and allows us to start the stimulation of the ovaries at any part of the cycle; there is no difference in oocyte number if stimulation is started in the luteal phase or the follicular phase. Currently, RSCOS is mainly performed in emergency oocyte cryopreservation in cancer patients (63–66), and less frequently in elective oocyte cryopreservation (67). New avenues will be explored in IVF patients. An example is an ongoing **late follicular phase stimulation start** project being conducted in Brussels (68). It has been clearly shown that the probability of an oocyte to provide a euploid blastocyst is the same for eggs obtained from the luteal phase as those obtained from the follicular phase (69). Another approach, particularly for poor responders, is to use **dual stimulation**. This can be helpful in situations in which one might want to save time by performing two stimulation treatments in a single menstrual cycle. Interestingly, recent data obtained in poor responders undergoing dual stimulation suggest that luteal phase stimulation results in more oocytes retrieved compared with follicular phase stimulation (70).

Another protocol modification compatible with a freeze-all strategy is **progestin-primed ovarian stimulation (PPOS)**, where the GnRH antagonist is replaced by a progestin, which can also suppress pituitary function. This strategy has been explored in China, where medroxyprogesterone or micronized progesterone have been used for preventing premature LH surges in women undergoing controlled ovarian hyperstimulation for IVF, with promising results so far (71, 72); however, longer-term safety data are required before this new strategy can be embraced.

#### Suboptimal Pituitary Response to GnRH Agonist Trigger

An important pitfall of the GnRH agonist trigger ovarian stimulation protocol is that the endogenous luteinizing hormone (LH) rise can be suboptimal in some patients (post-trigger LH ≤15 mIU/mL at 12 h post trigger). The LH level on the day of GnRH agonist trigger appears to be the most useful marker for predicting a patient's risk of having a suboptimal response; for example, 25% of patients with undetectable LH levels on the day of trigger show a suboptimal surge, compared with 2.7% of the overall population of women undergoing IVF-ICSI cycles (73). A suboptimal pituitary response is also frequently observed in long-term contraceptive pill-users, significantly diminishing the mature oocyte yield (74). Use of a **dual trigger of GnRH agonist combined with human chorionic gonadotropin (hCG)** for final oocyte maturation has been successful in improving oocyte retrieval rates in GnRH agonist suboptimal responders (75, 76).

### Is the Freeze-All Strategy Cost-Effective?

The quality and likelihood of implantation of frozen and fresh embryos are similar (77–79); however, data regarding cost-effectiveness of a freeze-all approach are sparse. Moreover, published studies investigating this aspect did not adjust for ovarian response categories. Indeed, since women with high ovarian response may have improved clinical outcomes after the freeze-all approach compared to fresh transfer, additional direct costs of cryopreservation, additional medication and subsequent FET cycles may be compensated by the more favorable success rates. In a study from Italy, there was no difference in cost between fresh blastocyst transfer and the freeze-all strategy (per live birth); the extra costs of vitrification, endometrial priming, and monitoring were offset by the fewer embryo transfer procedures needed, due to the efficiency of the freeze-all strategy (80). Obstetric and neonatal costs were not measured in this study. The cost-effectiveness of the freeze-all strategy was also demonstrated in a Brazilian study, in which treatment costs per ongoing pregnancy were significantly lower in freeze-all vs. fresh cycles (81).

When analyzing cost-effectiveness of the freeze-all and fresh transfer approaches, time-to-pregnancy should also be taken into account. Indeed, delay of embryo transfer in the freeze-all setting may heighten patient distress and anxiety accompanying an ART cycle. Nevertheless, even in a freeze-all setting, the delays between oocyte retrieval and embryo transfer can be kept to a minimum, since several retrospective trials have shown that FET can be performed as soon as the oncoming cycle after oocyte pick-up, without compromising success rates (82–88). Finally, when comparing health economic aspects of a freeze-all strategy and fresh embryo transfer, one has to take into account that adverse obstetric and neonatal outcomes such as preterm delivery and low birth weight may have a long-term impact on cost; therefore, modeling is required to inform cost-effectiveness over an extended time horizon (89).

### Safety of Reproductive Cryopreservation

Observations about the outcomes of reproductive cryopreservation in terms of effects on pregnancies and on neonates reveal several consistent findings. An increased risk of placental problems (such as placenta accreta), pregnancy-induced hypertension, and pre-eclampsia has been observed following FET (90–93), although a recent large randomized controlled trial found no differences in pre-eclampsia or hypertensive disorders between eFET and fresh embryo transfer when eFET was performed in the natural cycle (29). Compared with infants born after fresh embryo transfer, those born after FET had a lower risk of prematurity according to some studies (94–97), although other studies did not find any difference (98, 99). Infants born after FET have also been reported to have a lower risk of small-for-gestational age (96, 100, 101), and a higher birthweight (102) than those born after fresh embryo transfer. Less consistent findings for FET vs. fresh embryo transfer outcomes include more (90), similar (101), or fewer (103) perinatal/neonatal deaths, and lower monozygotic twinning rates (104).

A recent meta-analysis provides new evidence about whether there are differences in terms of neonatal outcome following fresh or frozen embryo transfer cycles (Table 2) (105). Frozen cycles are associated with a lower risk of prematurity or having low birthweight, but a higher risk of high birthweights and, importantly, a higher risk also for hypertensive disorders of pregnancy (105, 106). For some neonatal outcomes there is no significant difference between frozen and fresh transfer strategies: antepartum hemorrhage, admission to the neonatal intensive care unit, congenital abnormalities, and perinatal mortalities (105).

**Table 2:** Outcomes of FET vs. fresh embryo transfer in mothers and neonates.

**Favors FET**		**Favors fresh embryo transfer**		**No difference**	
**Outcome**	**FET vs. fresh embryo transfer RR (95% CI)**	**Outcome**	**FET vs. fresh embryo transfer RR (95% CI)**	**Outcome**	**FET vs. fresh embryo transfer RR (95% CI)**
Small for gestational age	0.61 (0.56–0.67)	Large for gestational age	1.54 (1.48–1.61)	Antepartum hemorrhage	0.82 (0.66–1.03)
Low birthweight (<2,500 g)	0.72 (0.67–0.77)	High birthweight (>4,000 g)	1.85 (1.46–2.33)	Admission to NICU	0.99 (0.84–1.18)
Very low birthweight (<1,500 g)	0.76 (0.69–0.82)	Very high birthweight (>4,500 g)	1.86 (1.58–2.19)	Congenital abnormalities	1.01 (0.87–1.16)
Preterm delivery (<37 weeks)	0.90 (0.84–0.97)	Hypertensive disorders of pregnancy	1.29 (1.07–1.56)	Perinatal mortality	0.92 (0.78–1.08)
Very preterm delivery (<32 weeks)	0.85 (0.74–0.97)	

*Summary results from cumulative meta-analyses. Adapted from Maheshwari et al. (105)*.

*CI, confidence interval; FET, frozen embryo transfer; NICU, neonatal intensive care unit; RR, relative risk*.

Higher birthweight associated with FET has been observed in large epidemiological studies in the UK (100), and in Scandinavia (92, 107), and has been further analyzed in meta-analyses by Pinborg et al. (108, 109). However, it is not clear whether the FET process itself contributes to higher birthweight or if other factors are involved. Importantly, birthweight could be influenced by the endometrial preparation with estrogens for FET, as no difference in birthweight was seen when embryos were transferred in a natural cycle (29). The increased risk of being born large for gestational age after FET has been observed both after vitrification and after slow freezing, and cryostorage duration of vitrified blastocysts does not appear to affect pregnancy and neonatal outcomes (102). However, further research is required to understand the physiological mechanisms that contribute to the larger birthweight associated with FET.

The use of estrogens in endometrial priming protocols during artificial FET cycles has been proposed as an explanation for the increased risk of pre-eclampsia associated with FET (15), based on contrasting findings from two large randomized controlled trials. In the first study, 1,508 women with PCOS were randomized to fresh embryo transfer or to eFET, with eFET cycles performed after estradiol valerate priming (25). Women in the eFET group had a higher rate of pre-eclampsia than those who underwent fresh embryo transfer (4.4 vs. 1.4%; *P* = 0.009) (25). A separate study (*N* = 2,157; in which women with PCOS were excluded) found no differences in pre-eclampsia or hypertensive disorders between eFET and fresh embryo transfer when eFET was performed in the natural cycle (29). Recent work has demonstrated that programmed cycles for FET were associated with higher rates of pre-eclampsia (110). The authors observed that programmed FET cycles, during which the corpus luteum is absent, are associated with impairment of the expected pregnancy-associated increase in central arterial compliance, probably because of absence of circulating vasoactive factors produced by the corpus luteum.

Another consideration is the direct effect of the cryopreservation process itself. According to published data, oocyte survival rates in young women who request planned oocyte cryopreservation for non-medical reasons are reassuringly high, with rates of 90% and higher (111). Furthermore, survival rates of oocytes vitrified using closed methods have been consistently higher than those of oocytes vitrified using open methods (112). Nevertheless, women should be informed that survival rates are age-dependent, reflecting age-related quality decline, and women above the age of 35 years should be counseled that 20% or more of vitrified oocytes may not survive the warming process (111).

One area of study is whether there is an epigenetic effect of the cryopreservation process. According to the hypothesis of the developmental origins of health and disease (DOHaD), cryopreservation of oocytes or embryos may disturb epigenetic mechanisms and by doing so, influence embryonic gene expression, which could result in altered development of the placenta and fetus in the early embryonic stages and cause changes in growth patterns and metabolic parameters. This could eventually lead to disease at later stages in life (113, 114). Hence, future follow-up studies of health in offspring conceived after oocyte and embryo cryopreservation are mandatory. Genome-wide analysis of placental miRNAs—important epigenetic regulators of gene expression—has demonstrated differentially expressed miRNAs in FET placentae compared with placentae from fresh embryo transfers, potentially contributing to increased birthweight and perinatal complications. There was no difference in expression between placentae from fresh embryo transfers vs. spontaneous pregnancies (115). The underlying mechanism is unclear, but these findings suggest this may be an important safety measure to explore in the longer term.

The impact of ART techniques on imprinting errors remains unclear, as the infertile population likely confers an independent risk factor for defects in expected epigenetic patterns (116). While some studies have observed an impact on bovine (117) and mouse embryos (118, 119), analysis of fresh vs. vitrified sibling human oocytes found that there was no difference in terms of maturation rates (i.e., epigenetic imprints) in the blastocysts after vitrified oocytes compared to fresh oocytes (120). Consistent with this finding, data from 1,027 children born after oocyte cryopreservation and 1,224 from fresh oocytes suggest that there is no increased risk of adverse obstetric and perinatal outcomes in children conceived with vitrified oocytes (121).

## Freeze-All for All Patients?

Despite the recent steady increase in the use of eFET as a component of ART, debate continues about whether eFET should be a standard treatment option available to the overall IVF population or if it is important to identify patient subgroups who are most likely to benefit from such an approach.

The eFET strategy has been compared with fresh embryo transfer in several large randomized controlled trials (22, 23, 25, 27, 29, 30); among these, a significantly superior benefit for FET over fresh embryo transfer has only been proven in women with PCOS, high responders, and in the setting of PGT-A (15).

Early positive results for FET appeared to support a shift to a freeze-all strategy for an increasing proportion of patients. A randomized single-center study of 103 first-time IVF patients in the US reported rates of ongoing pregnancies of 78% compared with 51% after cryopreservation cycles and fresh embryo transfers, respectively (*P* = 0.0072) (23). However, there were a number of limitations and biases: a small number of patients, the effect of co-interventions (dual triggering) was not considered, and there were abnormally high pregnancy rates in the cryopreservation cycles. A study from Brazil comparing the outcomes of freeze-all cycles (*N* = 179) and fresh transfer cycles (*N* = 351) also showed significant improvements in terms of implantation rates and clinical and ongoing pregnancy rates when a freeze-all strategy was applied (122). However, the population undergoing the freeze-all strategy also had significantly higher progesterone levels on the day of ovulation trigger, suggesting that the two study groups were not directly comparable (122).

Other studies do not support the concept that a freeze-all strategy is suitable for all patients. A retrospective study of 882 women aged 20–44 years undergoing their first or second IVF/ICSI cycle showed that there was no benefit on LBR of freeze-all vs. fresh transfer in normo-ovulatory women undergoing IVF (123). Patients with a risk of OHSS, high responders and women with high progesterone levels on the day of trigger were excluded from the study because those subgroups were already known to show improved outcomes with FET. Patients produced a normal oocyte yield (4–20 oocytes) in response to controlled ovarian stimulation and the results of embryo transfer showed that there were no differences for eFET vs. fresh embryo transfers in these normal responders in terms of implantation, clinical and ongoing pregnancies, and live births (123).

These findings have been confirmed recently in two large randomized controlled trials from China (*N* = 2,157) (29) and Vietnam (*N* = 782) (30). These studies show that for groups that are comparable in terms of ovarian stimulation protocol and dose, ovarian response, and late follicular progesterone levels, there is no benefit in LBR of eFET compared with fresh embryo transfer in normo-ovulatory women undergoing IVF. Importantly, in the Chinese study, the risk of women experiencing moderate or severe OHSS was significantly lower in the women who received the eFET strategy than in those who received fresh embryo transfers (0.6 vs. 2.0%; *P* = 0.005) (29).

A new meta-analysis of 11 randomized trials including 5,379 patients who underwent IVF/ICSI reported a significantly higher LBR with eFET compared with fresh embryo transfer in the overall IVF/ICSI population [risk ratio (RR), 1.12; 95% CI, 1.01–1.24], but no significant difference in CLBR (15). In subgroup analyses, the LBR benefit was only evident in hyper-responders (at high risk of developing OHSS; RR, 1.16; 95% CI, 1.05–1.28) and in PGT-A cycles (RR, 1.55; 95% CI, 1.14–2.10). There was no difference for LBR in normo-responders.

Beyond the potential risks associated with artificial cycle fresh embryo transfer in high responders, these findings suggest that in this population, there might be an impairment of endometrial receptivity due to a direct impact of high steroid levels, mainly progesterone, on endometrial maturation (47, 124).

The effect of FET on neonatal outcomes and the relative risks associated with frozen vs. fresh embryo transfers have been discussed previously in the section “Safety of Reproductive Cryopreservation.” These findings suggest that the obstetric background of the patient should also be considered when making a decision about a fresh transfer or a freeze-all strategy.

### Indications in Which Freeze-All Is Beneficial

Based on the findings reported above, there are two scenarios in which a freeze-all strategy is significantly superior to fresh embryo transfer.

#### PCOS Patients and OHSS Prevention

In high responders, in whom the risk of potentially life-threatening OHSS in response to ovarian stimulation in IVF is well-known, the benefit of a freeze-all strategy has been demonstrated in a large (*N* = 1,508) randomized controlled trial in China (25). The LBR after first embryo transfer was 49.3% for eFET vs. 42.0% for fresh embryo transfer (*P* = 0.004). Importantly, the frequency of OHSS was significantly lower in eFET cycles vs. fresh transfer cycles (1.3 vs. 7.1%; *P* < 0.001). These results support the recommendation of an eFET strategy in women with PCOS, in order to optimize the response whilst also minimizing the risk of OHSS.

#### PGT-A Programs

The development of PGT-A in the past years has been facilitated by the opportunity to use the eFET strategy for storing embryos for transfer in subsequent cycles. A successful fresh embryo transfer in this setting requires expanded blastocysts be available on the morning of day 5, and for PGT results to identify a suitable euploid embryo in time for transfer on day 6. The freeze-all strategy allows time for PGT results to be obtained for a whole cohort of embryos, which also results in a higher proportion of patients reaching embryo transfer. Transfer of a suitable euploid embryo is performed in a subsequent cycle. Indeed, there is increasing evidence that, in terms of implantation and ongoing pregnancy rates and clinical outcomes, FET in a non-stimulated cycle may be superior to performing a fresh embryo transfer in a stimulated cycle (122, 125–128). In a randomized controlled trial in 179 patients undergoing IVF treatment and PGT-A, ongoing pregnancy rates (80 vs. 61%; *P* = 0.03) and LBR (77 vs. 59%; *P* = 0.04) were significantly higher in the eFET group compared with those receiving fresh embryo transfer (27).

In addition to PCOS and PGT-A, there are other situations in which eFET can provide the opportunity to delay embryo transfer when conditions during the ovarian stimulation cycle may not be optimal for implantation.

#### Follicular Phase Progesterone Elevation

Progesterone increase at the end of stimulation, if above a certain threshold level (~4–5 nmol/L [~1.25–1.5 ng/mL]), led to a sharp decrease in implantation rates following fresh embryo transfer in some studies (47, 57), but this was not reported in others (129, 130). If all of the embryos are frozen and FET performed in a subsequent natural or endometrial preparation cycle, results have been reported to be better (57). As described above, the impact of high progesterone seems to be on the endometrium, only and, therefore, on implantation in the stimulation cycle whereas the quality of eggs collected for cryopreservation is not affected (58, 59).

#### Concomitant Endometrial or Tubal Pathology

The presence of endometrial polyps or a hydrosalpinx during the ovarian stimulation procedure is another scenario that can affect uterine receptivity (131, 132), and in the past would have resulted in an impaired or canceled ART cycle. With cryopreservation, the complication can be overcome by freezing the embryos, resolving the pathology, and transferring the frozen embryo in a subsequent cycle.

#### Adenomyosis

Results of a small study showed a non-significant trend to improved outcomes with FET in women with adenomyosis (133), but large randomized clinical trials are needed to confirm this hypothesis.

#### Slow Embryo Development

A successful embryo transfer and implantation requires embryo maturation to be at a synchronous stage with endometrium receptivity. In patients in whom embryo development does not reach blastocyst stage by day 5, transfer is delayed; implantation rates on day 6 are 15–18% lower compared with day 5 transfer (134). Alternatively, if embryos are frozen and transferred in the second cycle, outcomes will be improved compared with fresh transfer by avoiding the dys-synchrony between an endometrium that is too advanced vs. an underdeveloped embryo.

The freeze-all strategy has also allowed for the development of new ovarian stimulation protocols that optimize oocyte retrieval but do not allow for transfer within the same cycle—these include RSCOS, PPOS, and dual stimulation, as described above in the section “How Do We Optimize Mature Oocyte Yield and Oocyte Quality?” In these cases, cryopreservation is necessary because the endometrium will be out of phase for implantation within the same cycle.

## Optimizing the Frozen Embryo Transfer Cycle

### Methods of Freeze-All

The first successful pregnancies from frozen embryos in the 1980s used slow-freeze and rapid-thaw cryopreservation techniques. Slow freezing results in a liquid changing to a solid state, and technical issues—particularly with oocytes—such as intracellular ice formation and disrupted intracellular morphology led to low success rates and slow progress in the field, despite attempts to improve components of the process, including cryoprotectants, equilibration timing, cooling rates, and freezing devices [reviewed by Argyle et al. (44)]. More recently, vitrification methods have been developed to overcome these issues. Vitrification uses higher concentrations of cryoprotective additives and ultra-rapid cooling, lowering the risk of ice nucleation and crystallization to produce a non-crystalline amorphous solid. It is now established that vitrification is much more efficient than slow freezing, regardless of whether they are cleavage stage embryos or blastocysts (135), and that the stage of embryo development at which embryos are frozen does not have an impact on survival rates or implantation rates (78). Vitrification has made oocyte cryopreservation a reality and allowed it to become an established option for ART (44). The results of four randomized controlled trials (32, 136–138) showing that fertilization and pregnancy rates after IVF with vitrified oocytes were similar to those using fresh oocytes led to the adoption of oocyte cryopreservation in medical guidelines for ART (40).

### Role of Progesterone at Time of Implantation

Data from non-human studies and studies of spontaneous conception cycles and frozen-thawed embryo transfer cycles indicate that in the mid-luteal phase, there is a relatively narrow window of serum progesterone levels within which implantation is most likely to occur (5, 139, 140). In a prospective multicenter cohort study of 602 women undergoing IVF/ICSI fresh blastocyst transfers an optimal mid-luteal progesterone range of 150–250 nmol/L (47–79 ng/mL) secured the highest LBR (54%), compared to 42 and 38% at progesterone levels <150 nmol/L (<47 ng/mL) and >400 nmol/L (>126 ng/mL), respectively (5). For early luteal progesterone and pregnancy rate, there was a bigger difference: the highest pregnancy rate (73%) was achieved with early luteal progesterone of 60–100 nmol/L (19–31 ng/mL); outside this range, pregnancy rates were significantly lower, particularly at high levels of progesterone (>400 nmol/L [>126 ng/mL]: pregnancy rate 35%; difference −38%; *P* = 0.01). All women in this study received the same vaginal luteal support regimen.

Adequate circulating progesterone levels at embryo transfer are important to upregulate endometrial genes not only for successful implantation, but also during early pregnancy (141). Apart from regulating the window of implantation, progesterone increases endometrial vascularization and works as an immune modulator to securing the onward growth of the early implant (141). Hence, high rates of early pregnancy loss are seen when progesterone supplementation is suboptimal during artificial endometrial preparation (142). A series of three studies evaluating increasing levels of luteal support in an ovarian stimulation protocol of GnRH agonist trigger with fresh transfer illustrates the relationship between mid-luteal progesterone and pregnancy rates (Table 3) (1, 143, 144). Across the three studies, there was substantial decrease in early pregnancy loss and an increase in ongoing pregnancy rate as mid-luteal progesterone increased. Based on these collective findings, a lower cut-off level of around 75 nmol/L (24 ng/mL) progesterone at oocyte pick-up was defined to secure the highest clinical pregnancy rate (~40%), which is in line with the expected ongoing pregnancy rate at week 12 in a single embryo transfer program in those centers.

**Table 3:** Reproductive outcomes after embryo transfer in cycles with different levels of luteal phase progesterone support (1, 143, 144).

**References**	**Progesterone at OPU + 7 days, nmol/L (ng/mL)**	**Positive hCG per embryo transfer, %**	**Pregnancy loss, %**	**Clinical pregnancy, %**	**Luteal phase support**
Humaidan et al. (143)	39 ± 30 (12 ± 9)	29 (14/48)	79 (11/14)	6 (3/48)	Progesterone vaginal gel (90 mg/day)
Humaidan et al. (144)	74 ± 52 (23 ± 16)	48 (63/130)	21 (13/63)	38 (50/130)	hCG 1,500 IU on the day of OPU plus progesterone vaginal gel 90 mg per day
Humaidan et al. (1)	440 ± 25 (138 ± 8)	43 (47/110)	9 (4/47)	39 (43/110)	hCG 1,500 IU on the day of OPU and at OPU + 5 days plus progesterone vaginal gel 90 mg/day

*EPL, early pregnancy loss; GnRH, gonadotrophin-releasing hormone; hCG, human chorionic gonadotropin; OPU, oocyte pick-up; P, progesterone*.

Elevated progesterone in the late follicular phase has also recently become recognized as a factor that may impact implantation in fresh embryo transfer cycles and is known to have a negative impact on pregnancy rate (45–47). There is currently no evidence to suggest that results in FETs are affected by high progesterone levels in the follicular phase.

Whether serum measurements of progesterone can predict endometrial receptivity has been explored in a recent study in healthy women undergoing modeled endometrial cycles with varying intramuscular (IM) progesterone dosing (2.5, 5, 10, or 40 mg/day) after GnRH down-regulation and transdermal estradiol (145). Endometrial samples taken on day 10 of progesterone revealed apparently normal receptive histology in all except the lowest dose group, in which morphological delay was evident. Thus, most samples appeared to show a receptive endometrium. This was despite serum progesterone levels reaching the typical mid-secretory phase range only in the highest dose group. The same study looked at microarray analysis of endometrial gene expression as a measure of functional response to progesterone and found a significantly different pattern. Only patients that had the highest serum progesterone showed an endometrial gene expression profile comparable to natural cycle control patients, which is compatible with a receptive endometrium; a consistently altered functional response was seen in both of the lower dose groups (145). These results suggest that histology does not provide a reliable indication of endometrial receptivity, but gene expression profiles indicate that insufficient progesterone in the luteal phase leads to poor endometrial functionality.

### How Do We Prepare the Endometrium for FET?

#### Natural or Artificial Cycles for FET (NC-FET vs. AC-FET)

Once the decision to cryopreserve has been made, there is an ongoing debate about how to prepare the patient for embryo transfer, and whether a natural cycle or an artificial endometrial preparation cycle is preferred. Based on the published literature, the most common protocols used for FET preparation are a natural cycle with or without hCG trigger, or endometrial preparation with estrogen/progesterone hormone therapy with or without GnRH agonist suppression (146). A recent meta-analysis shows a non-significant trend in favor of natural cycles for better clinical and ongoing pregnancy rates and LBR [LBR odds ratio, 1.21; 95% CI, 0.96–1.51; *P* = 0.10] (18). The recently updated Cochrane systematic review also concluded that there is no difference in LBRs following different methods of endometrial preparation for FET (146). However, the quality of available evidence used in these analyses is poor, being derived predominantly from retrospective studies; therefore, prospective randomized studies are needed before any firm conclusions can be made about the superiority of one protocol over another.

A number of additional questions remain to be resolved: the optimal monitoring regimen for the natural cycle FET has not yet been determined; the routine used of luteal phase support in the natural cycle increases treatment burden, but it is not clear whether it is beneficial; in artificial cycles, the relative benefits of various routes of estrogen and progesterone administration need to be evaluated; the minimum duration of estrogen and progesterone supplementation also needs further clarification. Some of these questions are explored in studies described below.

#### What Is the Evidence for Giving Luteal Phase Progesterone Support for Embryo Transfer in a Natural Cycle?

A randomized controlled trial of 435 women undergoing embryo transfer in natural cycles demonstrated a significantly higher LBR group in the group who received vaginal progesterone (400 mg twice a day from the day of embryo transfer) compared with those who received no progesterone support (30 vs. 20%; *P* = 0.027) (147). In a retrospective cohort study of 228 consecutive women who received FET in modified natural cycles, those treated with progesterone vaginal gel (Crinone gel 8%; 90 mg/day from 2 days after hCG) experienced a significantly lower miscarriage rate (8.5 vs. 24.1%; *P* = 0.044) and achieved a significantly higher LBR (37.2 vs. 24.1%; *P* = 0.041) than the no progesterone group (148).

#### Is the Route of Administration of Progesterone Important for Artificial Endometrial Preparation?

In a planned interim analysis of a three-arm randomized controlled trial in women undergoing FET with different modes of progesterone replacement, vaginal progesterone alone was found to be inferior to protocols containing IM progesterone (149). A total of 645 FET cycles were randomized to: IM progesterone 50 mg per day; IM progesterone every 3 days with vaginal progesterone 200 mg per day; or vaginal progesterone 200 mg every 12 h. The group receiving only vaginal progesterone had a significantly higher miscarriage rate and a significantly lower ongoing pregnancy rate than the other two groups, and randomization to the vaginal progesterone group was discontinued as a result of these findings. The study is ongoing to compare the two IM progesterone protocols.

#### Optimizing Mid-luteal Progesterone

In the series of three studies evaluating increasing levels of progesterone support (1, 143, 144) [described above in the section “Role of Progesterone at Time of Implantation”], modified luteal phase support comprised vaginal progesterone gel 90 mg/day alone or with the addition of one or two doses of 1,500 IU hCG around day of oocyte pick-up, with corresponding increases in progesterone levels (Table 3). The optimum range of mid-luteal progesterone levels was achieved with the intermediate regimen of progesterone vaginal gel plus a single dose of hCG (144); the addition of a second hCG dose in the third study (1) resulted in a higher mid-luteal progesterone (440 nmol/L; 138 ng/mL) and a significant reduction in early pregnancy loss rates, whereas the ongoing pregnancy rate was similar to that seen in the previous study.

#### What Is the Impact of Serum Progesterone Levels in Estrogen/Progesterone Hormone Therapy Cycles for FET?

Results of a retrospective cohort study of 346 women who underwent FET after endometrial preparation with estradiol and progesterone vaginal gel 90 mg (Crinone) showed that using progesterone twice a day rather than once a day was associated with significantly better implantation rates (20.2 vs. 7.6%; *P* = 0.0001) and delivery rates (20.5 vs. 8.7%; *P* = 0.002), and significantly lower early pregnancy loss rates (43.7 vs. 67.4%; *P* = 0.014) (139).

In another study, low serum progesterone levels on the day of transfer were associated with lower ongoing pregnancy rates in a total of 211 oocyte recipients in a standard hormone therapy FET program with an artificial endometrial preparation cycle of oral or transdermal estradiol and vaginal micronized progesterone (400 mg/12 h from 5 days before embryo transfer) (142). Even though these patients all received the same regimen, there was a wide range of progesterone levels on the day of transfer, suggesting that vaginal progesterone was absorbed at different rates or to different extents among individual women. There was a corresponding variation in reproductive outcomes. Patients with the lowest serum progesterone (<29 nmol/L [9.2 ng/mL]) on the day of embryo transfer achieved significantly lower pregnancy rates (33%) than those with the highest levels (≥50 nmol/L [≥15.8 ng/mL]; 51%; *P* = 0.016); in fact, the most favorable results across all outcomes were achieved in the 3rd-quartile group (progesterone level range ~38–50 nmol/L [~11.8–15.7 ng/mL]) (142). These findings were supported by a recent retrospective study in which serum progesterone <32 nmol/L (<10 ng/mL) was associated with significantly lower pregnancy rates (34 vs. 48%; *P* = 0.04) and LBR (17 vs. 31%; *P* = 0.01) compared with progesterone ≥32 nmol/L (≥10 ng/mL) (150).

A new study—again in women undergoing a standard hormone therapy FET program, with transfer of high quality blastocysts—has also shown correlation between ongoing clinical pregnancy and progesterone levels (151). In agreement with Labarta et al. (142), the progesterone level for the best ongoing pregnancy rate (58% at week 12) was ≥35 nmol/L (≥11 ng/mL); for progesterone levels below this cut-off, ongoing pregnancy rate per embryo transfer was 44% (risk difference 14%, *P* = 0.02) (151).

## Conclusions

In recent years, the freeze-all strategy has emerged as an accepted and valuable alternative to fresh embryo transfer in ART, thanks to increasing demands for fertility treatment and improvements in cryopreservation techniques. The evidence overall indicates that outcomes of eFET are as good as those of fresh embryo transfer in the general population requiring fertility treatment, and in some populations better. This makes it an attractive option in a number of medical or elective scenarios in which it is advantageous to preserve oocytes or embryos and to defer embryo transfer until a later time.

In both fresh and frozen embryo transfers, several factors influence the success of ART. For some of these factors there may be room for optimization before embarking on treatment; however, maternal age is still the most important factor influencing reproductive outcomes in frozen as well as fresh embryo transfer; sperm DNA fragmentation and serum progesterone at peri-implantation are also important factors that are likely to be a focus for research and patient monitoring/screening during the coming years. Thus, recent data support the notion of a significant role for serum progesterone as a biomarker of endometrial receptivity in embryo transfer cycles, and a number of different strategies have been explored in the search for optimal endometrial preparation protocols.

In conclusion, cryopreservation in ART has already substantially changed the therapeutic landscape and continues to evolve as we learn more about ways to optimize the protocols and their applications to specific groups of patients. Going forward, more prospective randomized controlled trials are needed, indications for freeze-all need to be clearly defined, cryopreservation protocols need to be fine-tuned, cost-effectiveness studies are required, and societal aspects must be addressed. Last, but not least, the safety aspects of cryopreservation to both women and their offspring require further scrutiny and long-term assessment.

## Author Contributions

EB, MD, and PH contributed equally to the publication. All authors have been fully involved in the preparation of the manuscript at all stages from concept to final draft and approval and take accountability for all aspects of the work.

### Conflict of Interest

EB has received a research grant from Finox (part of the Gedeon Richter group); participated in advisory boards for Finox, MSD, and Roche Diagnostics; and participated in speakers bureaux and received honoraria from Ferring, Merck Serono, and MSD. MD has received lecture fees from Besins Healthcare, Cook Medical, Ferring, Gedeon Richter, and MSD. PH has received unrestricted research grants and honoraria from Ferring, Gedeon Richter, IBSA, Merck, and MSD. The reviewer GB declared a past co-authorship with one of the authors PH to the handling editor.
